# A Geroscience Perspective on COVID-19 – Joint Program from the American Aging Association (AGE) and The Gerontological Society of America

**DOI:** 10.1093/geroni/igaa057.1716

**Published:** 2020-12-16

**Authors:** Rozalyn Anderson

## Abstract

Faculty will focus on the biology of aging as a contributor to the vulnerability in COVID-19. Faculty will present the latest concepts and insights that will advance our ability to confront this global outbreak. Our goal for this session is to connect with the concept of Geroscience and how ideas from aging biology research can be incorporated to improve outcomes and informed practice. Although the emphasis is on biology, the goal is to provide insight in a manner that is readily accessible to researchers across the aging spectrum that they might translate these ideas in the face of a very real-world challenge.

